# Impact of maternal demographics on knowledge of exclusive breastfeeding among nursing mothers in Ifelodun local government, Nigeria

**DOI:** 10.4314/ahs.v23i2.79

**Published:** 2023-06

**Authors:** Odebode Aminat Adeola, Abdulraheem Adijat Mojisola, Yusuf Jamila

**Affiliations:** 1 Department of Counsellor Education, University of Ilorin, Nigeria; 2 Department of Health Promotion and Environmental Health Education University of Ilorin, Nigeria; 3 Counselling and Human Development Centre, Al-Hikma University, Ilorin, Nigeria

**Keywords:** Maternal demographics, exclusive breastfeeding, nursing mothers, Ifelodun local government area

## Abstract

**Background:**

Breastfeeding remains the recommended feeding plan for infants however; several factors seem to affect its knowledge and practice.

**Objectives:**

This study aimed at investigating the impact of maternal demographics of age, educational status, religious affiliation and number of parities on knowledge of exclusive breastfeeding among nursing mothers in Ifelodun Local government area, Nigeria.

**Methods:**

The study employed a descriptive survey method. A total of 206 nursing mothers in Ifelodun Local Government were selected through purposive and random sampling techniques. The participants responded to a researcher-designed questionnaire titled Knowledge of Exclusive Breastfeeding Questionnaire. The psychometric properties of the instrument were established. The data collected were analysed using both descriptive and inferential statistics. For the demographic data, percentage was employed while chi-square statistical tool was employed to test the hypotheses at 0.05 significant level.

**Results:**

Nursing mothers in Ifelodun Local Government Area have high knowledge of exclusive breastfeeding (N=76.8%). Also, maternal demographics of age (X2= 25P=0.03), educational status (X2= 62.72; P=0.00), religious affiliation (X2= 11.01; P=0.01) and number of parity (X2= 84.01; P=0.02) have significant impact on mothers' knowledge of exclusive breastfeeding.

**Conclusions:**

Nursing mothers in Ifelodun Local Government Area, have knowledge of exclusive breast feeding especially those who are older, literate, Christian; and multi-parous. Maternal demographics should be considered when educating nursing mothers on exclusive breastfeeding.

## Introduction

Breast milk is an ideal, natural, and protective food for newborns^1^. Given that prolonging people's lives (by reducing mortality) 2,3 and preventing disease (by reducing morbidity) are some goals of public health^4^, exclusive breastfeeding has been recognized as an efficient advance to the achievement of these goals^5^. In a study by Stuebe^6^, breastfeeding was found to be protective against sudden infant death syndrome by reducing the risk by 50% at all ages during infancy. Exclusive breastfeeding involves the infant feeding solely on breast milk for the first 6 months of life. This implies that by exclusive breastfeeding, the mother will not give any other food to the child except breast milk. Exclusive breastfeeding is particularly relevant in low-income countries such as Nigeria where there is high risk of infections.

Breastfeeding practices and attitudes are influenced by demographic, biophysical, social, cultural, and psychological factors, and knowledge level. Several studies have demonstrated that mothers' demographics such as age could affect mother's knowledge and practice of exclusive breastfeeding in the first six months of life^6^, ^7^. Previous studies have shown that knowledge of exclusive breastfeeding does not translate to practice^8^, ^9^. A study by Ekanem, Asuquo and Eyo10 on attitude of working mothers to exclusive breastfeeding in Calabar Municipality, Cross River State, Nigeria involved 138 mothers. It was found that breastfeeding was practiced exclusively by 28% while, 59.4% of the mothers practiced mixed feeding. It was concluded that the attitude of mothers towards practices of exclusive breastfeeding was negative. 10 Furthermore, Peterside^11^ reported on 134 women with age range 20 to 35; with 59.7% of the respondents having secondary education. Some 59.7% of the mother's knew the correct definition of exclusive breastfeeding while 19.4% had never heard of exclusive breastfeeding. A total of 80.6% of the mothers had heard about exclusive breastfeeding from health workers; and all mothers breastfed their babies within the first six month of life. In a similar study by Adeyinka, Falaye, Aremo and Adedeji^12^, 78.4% of the women interviewed were not aware of exclusive breastfeeding; and only 27% could give the correct definition of exclusive breastfeeding.

It is generally agreed that exclusive breastfeeding is beneficial; and there are concerns about the effects of artificial formulas^10^, ^11^, ^13^. Artificial feeding is associated with more deaths from diarrhea in infants in both rural and urban area13. Brest milk is the ideal for healthy growth and development of infants. Infants and children in Ifelodun Local government area are affected by poor health care, lack of portable water, malnutrition, and poverty. Several demographic variables may and may not affect mothers' knowledge of exclusive breastfeeding e.g., Religion^8^, age and educational attainment, marital status and number of children that have been nursed^10^, ^11^, ^12^, ^14^, ^15^, ^16^. This study focused on knowledge of exclusive breastfeeding among nursing mothers in Ifelodun Local government area.

## Key issues

The practice of exclusive breastfeeding is generally low in Nigeria^17^ compared to other African countries such as Sudan, South Sudan, Zambia, and Malawi. The practice of exclusive breastfeeding is very low in Nigeria with only 17% of children < 6 months being exclusively breastfed^18^. The low practice is frustrating the efforts of the Federal Ministry of Health and Social Services, UNICEF and WHO. This appears to be largely caused by lack of knowledge about exclusive breastfeeding. When babies are not breastfed exclusively, it leads to poor baby growth, poor cognitive and sensory development. It also contributes to poor health and infant mortality. Non breastfeeding mothers are prone to several chronic illnesses such as breast cancer among others. Although many studies have been conducted on breastfeeding^12^, ^15^, ^19^, the state of exclusive breastfeeding in the remains low among nursing mothers in the Ifelodun LGA. In particular, mothers give their babies water, pap and solid foods with the belief that it will make the baby strong. No studies had been done on the knowledge of exclusive breastfeeding among nursing mothers in Ifelodun Local government area. Thus, this study investigated the impact of maternal demographics on knowledge of exclusive breastfeeding from the views of nursing mothers in Ifelodun Local government area, Nigeria. The study was carried to answer the following research question: Do nursing mothers in Ifelodun Local government area, Nigeria have knowledge of exclusive breastfeeding? Four hypotheses were formulated and tested in this study:

1. Maternal age will not have significant impact on the knowledge of exclusive breastfeeding among nursing mothers in Ifelodun local government area.
2. Maternal educational status will not have significant impact on the knowledge of exclusive breastfeeding among nursing mothers in Ifelodun Local government area.
3. Maternal religious affiliation will not have significant impact on the knowledge of exclusive breastfeeding among nursing mothers in Ifelodun Local government area.
4. Maternal number of parities will not have significant impact on the knowledge of exclusive breastfeeding among nursing mothers in Ifelodun local government area.

## Materials and Methods

### Study Design and Setting

A descriptive research design of the survey type was adopted for this study. The study was carried out among nursing mothers in of Ifelodun Local government area between September and November, 2018 using quantitative methods. The population of the people in the Local government area^20^ was projected to be 276,700 making it the 4th most densely populated LGA in Ifelodun Local government area. Using EPI Info Version 7 with an expected frequency of 50% of nursing mothers with good knowledge and practice, and 90% confidence level and 6.0% margin of error^21^; a non-response of 10%; a sample size of 206 was obtained.

### Sample and Sampling Procedures

The multistage sampling procedure was employed to select samples for the study. At stage 1, purposive sampling technique was adopted to select 3 maternities and 2 general clinics that were largely populated where nursing mothers attended for post-natal care and child immunization. At stage 2, simple random sampling technique was employed to select nursing mothers from the selected maternities and clinics. This was done through balloting the names of the attendees from the attendance register in the clinics. The researcher continued the sampling until the sample size was achieved. Only consented nursing mothers were sampled for the study.

### Data Collection Tool and Procedure

A researcher-designed questionnaire was used to collect data for the study. The questionnaire was divided into two sections. Section A contained the demographics of the respondents such as age, educational status, religious affiliation, and number of children. Age was categorized as 18-27 years old, 28-37 years old and 38 years old & above. Educational status was categorized as literate and not literate. Religious affiliation was classified as Christianity, Islam and African Traditional Religion. Parity was categorized as 1-3 children, 4-6 children and 7 children & above. Section B consisted of 15 items based on the level of nursing mothers' knowledge of exclusive breastfeeding for instance, ‘I know that: Exclusive breastfeeding the baby with breast milk only”. Section B was based on Yes or No responses, on a scale of 1 and 2 and the cut-off point was 50%22. This follows that an aggregate mean score between 50% and above implies high level of knowledge of exclusive breastfeeding while an aggregate mean scores below 50% implies low level of knowledge of exclusive breastfeeding among nursing mothers in Ifelodun Local government; this assessment criteria has been used by previous researchers23, 24, 25 The questionnaire was validated by five experts in the field of Medicine, measurement, and evaluation. The reliability of the instrument was ascertained through test re-test reliability method which yielded a coefficient value of 0.85; the instrument was therefore adjudged suitable for this study.

### Data Analysis Plan

The data were manually checked for any error, entered to Epidata and edited before being moved to SPSS V. 22.0 for analysis. The demographic data were analysed using descriptive statistics of percentage and research question while Chi-square was used to analyse the impact of maternal demographics on knowledge of exclusive breastfeeding. A p< 0.05 was considered significant for the testing of hypotheses.

### Ethical Consideration

The purpose of the study was explained to the participants and their consents were sought; none of the respondents was forced to participate in the study. The researcher established rapport with them, gave appropriate instructions and clarification on the items to encourage respondents' accurate and prompt response to the instrument. Participants who were not interested were allowed to excuse themselves. The participants were assured that their responses were to be used solely for research purposes and that there is no harm involved in participating in the study. The participants were not asked to write their names or addresses to ensure anonymity. The questionnaires were thereafter collected immediately after completion to avoid loss.

## Results

### Demographic Information

A total of 206 copies of questionnaire were administered and 200 (97%) copies were returned. The demographic data were analysed using descriptive statistics as shown in table 1.

**Table 1:** Demographic Distribution of Respondents by Maternal Age, Educational Status, Religious affiliotion and Numberof Parity

Variables	F	%
Age:		
18-27 years	99	49.5
28-37 years	101	50.5
38 years & Above	-	-
Total	200	100
Educational Status:		
Literate	106	53.0
Not Literate	94	47.0
Total	200	100
Religion		
Christianity	113	56.5
Islam	80	40.0
ATR	7	3.5
Total	200	100

Number of Children:		14.0
1-3	28	86.0
4-6	172	-
7 & Above	-	
Total	200	100

The analysis revealed that 99 (49.5%) of the respondents were between 18-27 years old, 101 (50.5%) of the respondents were between 28-37 years old, none of the respondents were between 38 years old and above. The rest of the demographic characteristics are shown in table 1.

### Knowledge of exclusive breastfeeding among nursing mothers in Ifelodun Local government area, Nigeria

The cut-off points for taking decision on the knowledge level of exclusive breastfeeding among mothers in Ifelodun Local government area is 50% and all the 15 items have mean scores above the cut-off also, with an aggregate mean of 76.8%. It is concluded that nursing mothers in Ifelodun Local government area have knowledge of exclusive breastfeeding.

### Hypotheses Testing

Four null hypotheses were formulated and tested for this study. The hypotheses were tested using chi-square statistical methods at 0.05 level of significance.

### Hypothesis One

Maternal age will not have significant impact on the knowledge of exclusive breastfeeding among nursing mothers in Ifelodun Local government area

As a test of the hypothesis that maternal age will not have any significant impact on the knowledge of exclusive breastfeeding among nursing mothers in Ifelodun Local Government Area, an independent sample t-test was used. This test was found to be statistically significant, (tobt(1) = 25.8, p < .05). This result suggests that a significant impact exists in the respondents' knowledge of exclusive breastfeeding based on maternal age; hence, the hypothesis is rejected. The respondents between ages 28 and 37 years (M = 92.3, SD = 8.7) caused the significant impact of maternal age on the knowledge of exclusive breastfeeding among nursing mothers in Ifelodun Local government area.

### Hypothesis Two

Maternal educational status will not have significant impact on the knowledge of exclusive breastfeeding among nursing mothers in Ifelodun Local government area.

The test of the hypothesis that maternal educational status will not have any significant impact on the knowledge of exclusive breastfeeding among nursing mothers in Ifelodun Local government area, an independent sample t-test was used. This test was found to be statistically significant (tobt (1) = 62.72, p < .05). This result indicates that a significant impact exists in the respondents' knowledge of exclusive breastfeeding based on maternal educational status; hence, the hypothesis is rejected. The respondents who are literate (M = 98.7, SD = 7.3) caused the significant impact of maternal educational status on the knowledge of exclusive breastfeeding among nursing mothers in Ifelodun Local government area.

### Hypothesis Three

Maternal Religious affiliation will not have significant impact on the knowledge of exclusive breastfeeding among nursing mothers in Ifelodun Local government area.

As a test of the hypothesis that maternal religious affiliation will not have any significant impact on the knowledge of exclusive breastfeeding among nursing mothers in Ifelodun Local Governme nt Area, an independent sample t-test was used. This test was found to be statistically significant, tobt (2) = 84.01, p < .05 This result illustrates that a significant impact exists in the respondents' knowledge of exclusive breastfeeding based on maternal religious; hence, the hypothesis is rejected. The respondents practicing Christianity (M = 107.4, SD = 5.6) caused the significant impact of maternal religious affiliation on the knowledge of exclusive breastfeeding among nursing mothers in Ifelodun Local Government Area.

### Hypothesis Four

Maternal number of parity will not have significant impact on the knowledge of exclusive breastfeeding among nursing mothers in Ifelodun Local government area.

As a test of the hypothesis that maternal number of parity will not have any significant impact on the knowledge of exclusive breastfeeding among nursing mothers in Ifelodun Local government area, an independent sample t-test was used. This test was found to be statistically significant, tobt(1) = 11.01, p < .05. This result suggests that a significant impact exists in the respondents' knowledge of exclusive breastfeeding based on maternal number of parities; hence, the hypothesis is rejected. The respondents who have 1-3 children (M = 100.3, SD = 7.7) caused the significant impact of maternal number of parities on the knowledge of exclusive breastfeeding among nursing mothers in Ifelodun Local government area.

## Discussion

This study has shown that mothers in Ifelodun Local government area have knowledge of exclusive breastfeeding. They know that exclusive breastfeeding prevents breast cancer, helps baby with teething problems, is cheap, available, and healthy, relieves the baby of bodily pains, and makes the baby grow normally. This finding tallies with Peterside^11^ who showed that mothers were knowledgeable about exclusive breastfeeding. Similarly, this finding is consistent with Agu and Agu^15^ who found that 87% of mothers in a rural area had knowledge of exclusive breastfeeding. However, this finding is inconsistent with Adeyinka, Falaye, Aremo and Adedeji^12^, and Gigante, et al^27^ who found that mothers in Nigeria and Ghana had limited knowledge of exclusive breastfeeding. This may be due to publicity of exclusive breastfeeding to Nigerian society through health personnel.

Hypothesis one, which stated that age will not have significant impact on the knowledge of exclusive breastfeeding among nursing mothers in Ifelodun Local government area, was rejected. This means that maternal age has significant impact on the knowledge of exclusive breastfeeding among nursing mothers in Ifelodun Local government area. In this study, nursing mothers who were between 28-37 years old had higher knowledge of exclusive breastfeeding. This finding is in line with Qui et al.28, Dia de Oliveira et al^29^, Avery et al^30^, Mundagowa, Chadambuka, Chimberengwa and Mukora-Mutseyekwa^31^ which showed that mothers aged below 25 years oldin Gwanda district in Zimbabwe, did not have knowledge of exclusive breastfeeding. Similarly, this finding is in line with Agu and Agu^15^ found significant difference in the knowledge of exclusive breastfeeding among mothers in a rural population based on age. This may be due to the fact that older nursing mothers have more experience on exclusive breastfeeding due to increase in age.

Hypothesis two, which stated that educational status will not have significant impact on the knowledge of exclusive breastfeeding among nursing mothers in Ifelodun Local government area, was rejected. This means that educational status has significant impact on exclusive breastfeeding knowledge among nursing mothers in Ifelodun Local government area. In this study, nursing mothers who were literate had higher knowledge of exclusive breastfeeding. This finding is like Adeyinka, Falaye, Aremo and Adedeji^12^ who found that literate nursing mothers had higher knowledge of exclusive breastfeeding. Similarly, the study is in line with Odebode, Okesina and Ola-Alani^19^ and Mundagowa, Chadambuka, Chimberengwa and Mukora-Mutseyekwa^31^ work which showed significant difference based on educational status. This might be due the fact that literate mothers are likely to be more exposed to sources of information such as social media, television, newspapers. Hence, they are likely to be more knowledgeable than the illiterate.

Hypothesis three which stated that religious affiliation will not have significant impact on the knowledge of exclusive breastfeeding among nursing mothers in Ifelodun Local government area, was rejected. This means that religious affiliation has significant impact on the knowledge of exclusive breastfeeding among nursing mothers in Ifelodun Local government area. In this study, nursing mothers who were Christian had higher knowledge of exclusive breastfeeding. This finding tallies with Adebayo, Leshi and Sanusi8 and Erick et al^14^ whose studies showed that knowledge of exclusive breastfeeding is high among certain religious adherents than others. This finding could be due to the method of worship and programmes organized in the various religious organizations. For instance, Christian nursing mothers are likely to receive training on exclusive breastfeeding to which other religious adherents are not exposed.

Hypothesis four, which stated that parity will not have significant impact on the knowledge of exclusive breastfeeding among nursing mothers in Ifelodun Local government area was rejected. In this study, nursing mothers with 4-6 children, have higher knowledge of exclusive breastfeeding. This finding is in support of Ado, Bruce and Owu7 whose showed that the more mothers have children, the more knowledgeable they are about exclusive breastfeeding. Similarly, this study is in line with Khasawneh and Khasawneh^26^ who found that mothers with multi-parity had higher knowledge of exclusive breastfeeding than those with less parity. It is possible that mothers with more children have higher experience of breastfeeding.

## Limitations

This study is quantitative in nature in which the participants responded to a structured questionnaire. The study also sampled only 206 nursing mothers in a Local Government Area. Therefore, the findings may not be generalized to all nursing mothers in other regions in the country. Since the research is descriptive, no causal relationship could be inferred; the study only assessed the relative impact of maternal age, educational status, religious affiliation and number of parities on the respondents' knowledge of exclusive breastfeeding and not the relationship between the moderating variables and the knowledge of exclusive breastfeeding of nursing mothers.

## Conclusion

Nursing mothers, in Ifelodun Local Government Area, have knowledge of exclusive breast feeding especially those who are older, literate, Christian and multi-parous. Maternal demographics should be considered when educating nursing mothers on exclusive breastfeeding. Studies with a larger sample size; and employing mixed methods are recommended.

## Figures and Tables

**Table 2:** Mean and rank order of knowledge of exclusive breastfeeding

Item No.	I know that:	%	Rank
15.	Exclusive breastfeeding prevents breast cancer	89.0	1^st^
14.	Exclusive breastfeeding helps baby with teething problems	88.0	2^nd^
12.	Exclusive breastfeeding is cheap, available and healthy	86.0	3^rd^
9.	Exclusive breastfeeding relieves the baby of bodily pains	85.0	4^th^
8.	Colostrum in the breast provides nutrients for the baby	84.0	5^th^
6.	Exclusive breastfeeding makes the baby grow normally	82.0	6^th^
1.	Exclusive breastfeeding the baby with breast milk only	80.0	7^th^
11.	Exclusive breastfeeding promotes mother-baby bonding	77.0	8^th^
13.	Exclusive breastfeeding caters for all aspects of the baby's health	74.0	9^th^
10.	Exclusive breastfeeding protects the baby from infection	70.0	10^th^
2.	Exclusive breastfeeding should start immediately	69.0	11^th^
7.	Exclusive breastfeeding serves as birth control	68.0	12^th^
4.	Exclusive breastfeeding should be practiced for the first six months of life	67.0	13^th^
5.	Exclusive breastfeeding is sufficient for the baby during the first six months of life	67.0	13^th^
3.	I should not give not give the baby water for the first six months of life	66.0	15^th^
	**Aggregate**	**76.8**	

**Table 3:** Chi-square (χ^2^) showing the analysis of impact of maternal age on respondents' knowledge of exclusive breastfeeding

Age	Knowledge			Total	df	Cal. X^2^-value	Cal. Sig. (2sided)	Decision
		High	Low					
18-27 years old	Observed	34(34.3%)	65(65.7%)	99				
	Expected	13.4	85.6	99.0				
								**H_01_**
	Observed	89(88.1%)	12(11.9%)	101	1	25.8*	0.03	**Rejected**
	Expected	92.3	8.7	101.0				
28-37 years old	Observed	123	77	200				
	Expected	105.7	94.3	200.0				
Total								

* p**<0.05**

**Table 4:** Chi-square (χ^2^) showing the analysis of impact of maternal religious affiliation on respondents' knowledge of exclusive breastfeeding

Religion	Knowledge			Total	df	Cal. X^2^-value	Cal. Sig. (sided)	Decision
		High	Low					
Christianity	Observed	100(88.5%)	13(11.5%)	113				
	Expected	107.4	5.6	113.0				
								**H_03_**
Islam	Observed	53(66.25%)	7(33.75%)	80	2	84.01*	0.01	**Rejected**
	Expected	68.7	11.3	80.0				
ATR	Observed	1(14.3%)	6(85.7%)	7				
	Expected	2.2	4.8	7.0				
Total	Observed	154	46	200				
	Expected	178.3	21.7	200.0				

* p**<0.05**

**Table 4:** Chi-square (χ^2^) showing the analysis of impact of maternal educational status on respondents' knowledge of exclusive breastfeeding

Educational	Knowledge			Total	df	Cal. X^2^-value	Cal. Sig. (2-sided)	Decision
Status		High	Low					
Literate	Observed	96(90.6%)	10(9.4%)	106				
				106.0				
	Expected	98.7	7.3					**H_02_**
Not Literate				94	1	62.72*	0.00	**Rejected**
	Observed	35(37.2%)	59(62.8%)	94.0				
Total	Expected	4.5	89.5	200				
				200.0				
	Observed	131	69					
	Expected	103.2	96.8					

* p**<0.05**

**Table 5:** Chi-square (χ^2^) showing the analysis of impact of maternal number of parity on respondents' knowledge of exclusive breastfeeding

Number of Parity	Knowledge			Total	df	Cal. X^2^-value	Cal. Sig. (2-sided)	Decision
		High	Low					
1-3 children	Observed	50(46.3%)	58(53.7%)	108				
				108.0				
	Expected	100.3	7.7					**H_04_**
				92	1	11.01*	0.02	**Rejected**
4-6 children	Observed	70(76.1%)	22(23.9%)	92.0				
	Expected	87.	4.9	200				
				200.0				
Total	Observed	120	80					
	Expected	187.4	12.6					

* p**<0.05**
